# Children’s Power of Food Scale: Turkish validity and reliability study

**DOI:** 10.1017/S1368980021003773

**Published:** 2021-12

**Authors:** Gülsüm Şahin-Bodur, Alev Keser, Mehtap Akçil-Ok, Emine Nüket Ünsal, Onur Akın

**Affiliations:** 1 Department of Nutrition and Dietetics, University of Ankara, Fatih Street, No: 197/A, Ankara 06100, Turkey; 2 Department of Nutrition and Dietetics, University of Başkent, Ankara, Turkey; 3 Gulhane Training and Research Hospital, Ankara, Turkey

**Keywords:** Power of Food, Hedonic hunger, Childhood obesity, Children, Adolescents

## Abstract

**Objective:**

The aim of the present study was to validate the Turkish version of the Children’s Power of Food Scale (C-PFS-T) after translation of the original version.

**Design:**

The data were collected via face-to-face interviews using the C-PFS-T and a socio-demographic information form. BMI was calculated by dividing body weight by the square of the height. After the adaptation of the scale to Turkish language, validity and reliability analysis were conducted for the C-PFS-T.

**Setting:**

Gülhane Training and Research Hospital Department of Child Health and Diseases Nutrition and Diet Unit in Ankara.

**Participants:**

This research was conducted with volunteer children and adolescents between the ages of 9 and 16 years (*n* 268).

**Results:**

It was concluded that the 15-item C-PFS-T was collected under three factors as in the original version of the child version. Cronbach’s *α* coefficient was found to be 0·878 for the scale. The confirmatory factor analysis results showed the acceptability and applicability of adapting the version of the C-PFS-T in terms of *χ*^2^/df (= 3·816), adjusted goodness-of-fit index (AGFI = 0·931), root mean square error of approximation (RMSEA = 0·082) and goodness-of-fit index (GFI = 0·852) fit indices. C-PFS-T total score’s median value of obese group wasn’t substantially different from normal weight group.Conclusions:

It was concluded that the Turkish version of the C-PFS, which provides an assessment of the hedonic hunger status of children and adolescents with fifteen items and threesubdimensions, has sufficient reliability and validity to be applied to these subjects.

The rapid rise in childhood obesity is one of the most serious public health issues of the 21st century, with the number of children and adolescents affected by obesity increasing more than tenfold from 11 million in 1975 to 124 million in 2016^(1)^. In Turkey according to 2010 data from the National Turkey Nutrition and Health Survey, 8·2 % of children in the 6–18 age group are obese and 14·3 % are overweight^(2)^. According to the Turkey Healthy Nutrition and Active Life Program study, 9·8 % of adolescents between the ages of 12 and 14 years are obese^(3)^. A meta-analysis showed that the obesity prevalence in children and adolescents between the ages of 5 and 19 was 5·7 %, and the prevalence in 2010–2015 increased 11·6-fold compared to that between 1990 and 1995^(4)^.

Obesity arises from a combination of exposure of the child to an obesogenic environment, and inadequate behavioural and biological responses to that environment^(5)^. In an obesogenic environment, hedonic hunger, which involves thoughts, feelings, and impulses related to food and appetitive motives, develops along with consumption of highly palatable foods^(6,7)^. Mechanisms contributing to the emergence of hedonic hunger include senses that perceive taste, smell, texture, and even sound, and these factors result in a subjective feeling of pleasure causing the individual to choose one food or another^(8)^. In some susceptible groups, frequent exposure to highly palatable foods may enhance hedonic processes even when they are not hungry, leading to overeating and weight gain. Thus, a tool that measures individual differences in appetite-related thoughts, feelings and motivations in environments where plentiful palatable foods are constantly available can be useful for identification of groups susceptible to the development of obesity^(9)^. Yet, the number of scales developed for this purpose for childhood is quite insufficient in Turkey and the rest of the world.

The Power of Food Scale (PFS) was developed by Cappelleri (2009) to evaluate the sense of being controlled by food and the psychological impact of being in a food-rich environment, regardless of food consumption^(6)^. The Children’s Power of Food Scale (C-PFS), which consists of fifteen items, was revised by Lowe to reflect the developmental levels of school-age children and to increase understanding of this group^(10)^.

In the literature, although the number of validation studies using the adult version of the PFS is quite high^(6,11,12)^, there are a limited number of studies using the child version of the scale^(13,14)^. To date, there are no valid and reliable questionnaires for appropriately assessing and measuring hedonic hunger in Turkey. For this reason, the aim of the present study was to validate the Turkish version of the C-PFS after translation of the original version (English).

## Methods

### Design and participants

This was a cross-sectional study conducted to analyse the validity and reliability of the C-PFS for the Turkish population.

It was conducted with volunteer children and adolescents between the ages of 9 and 16 years who were presented to Gülhane Training and Research Hospital Department of Child Health and Diseases Nutrition and Diet Unit between July 2019 and February 2020. The study did not include those on drugs that affect food consumption, those with chronic diseases or those who were underweight. For the Turkish validity and reliability study, it is recommended to apply the scale to participants numbering at least 5–10 times the number of items^(15)^. Accordingly, this scale with fifteen items should be applied to at least 75–150 individuals. The study was performed with 268 children and adolescents.

### Data collection form

The data started to be collected, the purpose was explained by giving information about the research to the participants and their families, and those who agreed to take part were included in the research. The data were collected via face-to-face interviews using the C-PFS and a socio-demographic information form (age, gender and educational status) (Table 1). The researchers obtained the body weight measurements using a Tanita BC-545N body analyser and height measurements using a stadiometer in accordance with the recommended technique. BMI was calculated by dividing body weight (kg) by the square of the height (m^2^), and those with a BMI ≥ 15·0–84·9 percentile were considered normal-weighted and those with one ≥ 95·0 percentile obese^(16)^ according to age and gender. The administration of these interviews took 10–15 min.

**Table 1:** Socio-demographic characteristics of the individuals

Characteristics	*n*	%
Gender		
Female	156	58·2
Male	112	41·8
Status of education		
Primary school	16	6·0
Middle school	109	40·6
High school	143	53·4
BMI		
Normal weight	113	42·2
Obese	155	57·8
Total	268	100·0
Age (years)		
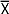	13·3	
sd	2·22	
Median	14·0	

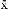
 : mean.

### The original instrument Children’s Power of Food Scale

The C-PFS consists of fifteen items and is answered using a five-point Likert scale: 1 = do not agree at all; 2 = agree a little; 3 = agree somewhat; 4 = agree; 5 = strongly agree)^(17)^.

The C-PFS questions the differences in the appetite response given to food in three different levels in an environment where the individual is close to the food. According to Lowe *et al.* (2009), the subdimensions of the original scale are as follows: Factor 1, food available, the fact that the food is not physically present in the environment but can be easily accessed, covers items 1, 2, 5, 10, 11 and 13; Factor 2: food present, the food is present in the environment but not yet tasted, covers items 3, 4, 6 and 7; Factor 3: food tasted, the taste of the food in the environment but not consumed completely, covers items 8, 9, 12, 14 and 15^(9)^. The C-PFS total and subdimension scores are obtained by summing the item scores and dividing the sum by the number of items. Higher scores indicate that the individual is more sensitive to the food-rich environment and is more controlled by foods psychologically^(6,9)^.

### The translation procedure – Turkish version of Children’s Power of Food Scale

The researchers obtained permission from the author of the scale to adapt it to Turkish. The standard translation-back-translation method was used to adapt the C-PFS to Turkish (C-PFS-T). For this purpose, five faculty members (four working in Ankara University Department of Nutrition and Dietetics and one in Boğaziçi University School of Foreign Languages) with good levels of English and Turkish were contacted. The scale was first translated from English, its original language, into Turkish, and whether it matched the original was checked by five faculty members working in the field of Nutrition and Dietetics. Approximately a week later, the scale was translated from Turkish to English and from English to Turkish. At all these stages, the faculty members worked independently. The final version of the scale was created by evaluating the consistency, meaning integrity and grammar by combining the translations done at the last stage.

The finalised scale was first applied to twenty individuals between the ages of 9 and 16 years and they were asked whether it was understandable. Their answers showed there was no need to make changes to the final version of the scale. These preliminary pilot data were not included in the research.

### Statistical analysis

Explanatory factor analysis was used to determine the numbers of items in the scale and to analyse its structural validity. Before this analysis was performed, the adequacy of the sample size was examined with the Kaiser–Meyer–Olkin (KMO) test (> 0·60)^(18)^, and Bartlett’s sphericity test (*P* < 0·05)^(19)^ was used to determine whether there was a correlation between the items that are a prerequisite for factor analysis.

Confirmatory factor analysis (CFA) was applied to analyse the compatibility of subdimensions with the original scale. Fit indicators such as the *χ*^2^ goodness-of-fit index, adjusted goodness-of-fit index (AGFI), root mean square residual, root mean square error of approximation (RMSEA) and goodness-of-fit index (GFI) were calculated.

Item analysis for internal consistency was performed and reliability coefficient/Cronbach’s *α* values were calculated. Cohen’s *κ* test, which is a statistical method that measures the reliability of agreement between two or more observers, was performed. Cohen suggested the *κ* agreement coefficients be interpreted as follows: values 0·00–0·20 as none to slight, 0·21–0·39 as minimal, 0·40–0·59 as weak, 0·60–0·79 as moderate and 0·80–0·90 as strong and almost perfect agreement ^(20)^.

The scale was reapplied to 18·6 % (*n* 50) of the sample number after a 2-week interval^(21)^for test–retest reliability (to reveal the scale’s invariance against time). Pearson’s correlation analysis was used for specifying the correlation between BMI and C-PFS-T total and subdimensions score.

Pearson’s correlation analysis for item-total score analysis of the scale was performed. This is an indicator of whether the items in the scale measure the desired quality. This value should be > 0·20, as close to 1 as possible and positive^(22)^.

SPSS v.25 with AMOS was used to analyse the validity and reliability of the C-PFS-T. In the statistical analysis, the significance level was accepted as *P* < 0·05.

## Results

A total of 268 children and adolescents participated. Their mean age was 13·3 ± 2·22 years and 58·2 % of them were female. The education level of more than half of the participants was high school. According to the BMI classification, 53·4 % of the children and adolescents were obese (Table 1).

In the present study, according to Bartlett’s sphericity test result, there was an acceptable relationship between the items of the C-PFS child version (*χ*^2^ = 1304·095; *P* = < 0·001). Moreover, according to the KMO test, the sample size was sufficient for factor analysis (KMO = 0·901 > 0·500).

As a result of the explanatory factor analysis, as in the original version of the scale, items were divided into three factors under the constraint of an eigenvalue > 1·00. As shown in Table 2, in the C-PFS-T, Factor 1 covers items 1, 2, 5, 10, 11 and 13; Factor 2 covers items 3, 4, 6 and 7; and Factor 3 covers items 8, 9, 12, 14 and 15 as in the original. Principal components analysis with Varimax rotation identified three factors and the scale was found to explain 54·73 % of the total variance. In addition, Factor 1 accounted for 37·2 %, Factor 2 accounted for 9·6 % and Factor 3 accounted for 7·8 % of the variance. Factor loadings of Factor 1, 2 and 3 subdimensions of the C-PFS-T were 0·463–0·760, 0·591–0·724 and 0·502–0·788, respectively (> 0·30). The explanatory factor analysis results of the C-PFS-T can be seen in Table 2.

**Table 2:** Explanatory factor analysis results of the C-PFS-T (*n* 268)

Items	Factor 1: Food available	Factor 2: Food present	Factor 3: Food tasted	Total scale
1. I think about food even when I’m not truly hungry	0·649			0·638
2. I get more pleasure from eating than I do from almost anything else.	0·686			0·614
5. I feel like food controls me instead of me controlling my food choices.	0·463			0·604
10. Sometimes, when I’m doing everyday activities, I get an urge to eat ‘out of the blue’ (for no good reason).	0·705			0·540
11. I think I enjoy eating a lot more than most other kids.	0·760			0·635
13. It seems like I have food on my mind a lot.	0·729			0·688
3. If I see or smell a food I like, I get a very strong desire to have some.		0·591		0·537
4. When I’m around a fattening food I love, it’s hard to stop myself from at least tasting it.		0·724		0·679
6. When I know a delicious food is available, I keep thinking about having some.		0·599		0·518
7. I love the taste of certain foods so much that I can’t avoid eating them even if they’re bad for me.		0·708		0·598
8. Just before I taste a favourite food, I get very excited.			0·561	0·654
9. When I eat delicious food, I focus a lot on how good it tastes.			0·788	0·671
12. Hearing someone describe a great meal makes me really want to have something to eat.			0·501	0·665
14. It’s very important to me that the foods I eat are as delicious as possible.			0·762	0·452
15. Before I eat a favourite food my mouth waters.			0·502	0·672
Eigenvalue	5·581	1·447	1·183	
Variance explanation percentage	37·206	9·646	7·887	54·73

### Confirmatory factor analysis

**Fig. 1:**
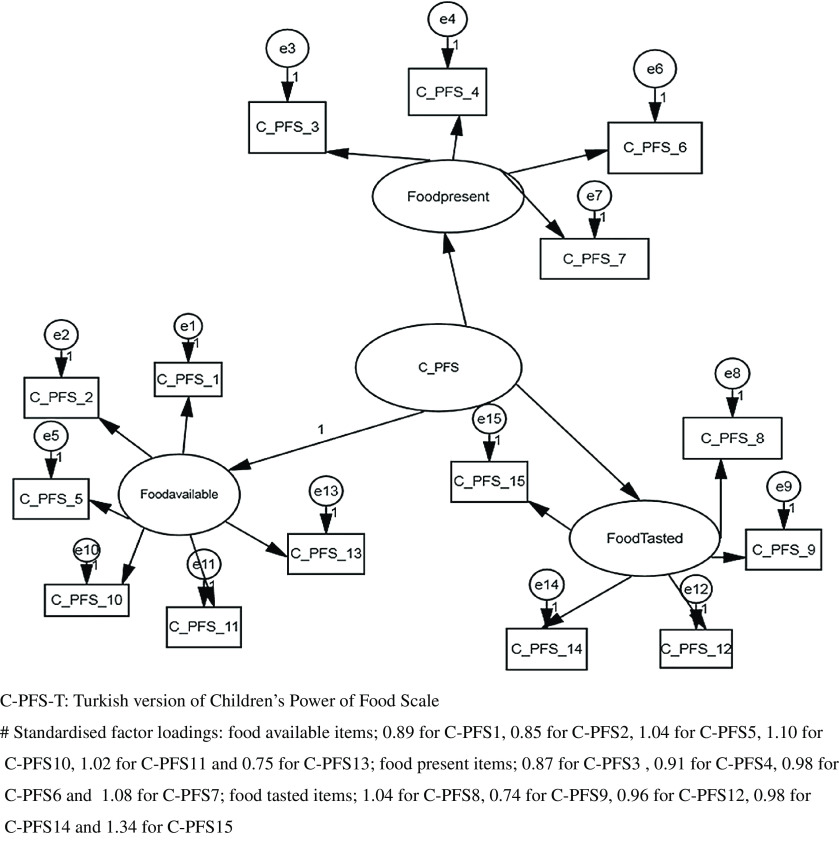
Confirmatory factor analysis of the C-PFS-T and three-factor model compliance diagram/path diagram^**#**^

Different indices can be used to evaluate the fit of a model. The fit indices of the model are shown in Table 3 and the three-factor model compliance diagram/path diagram is presented in Fig. 1. The *χ*^2^ goodness-of-fit index (*χ*^2^/df) was 3·816. In addition, the AGFI was 0·931. The RMSEA value was 0·082. The GFI value was 0·852. On the basis of the CFA, the standardised factor loadings of Factor 1 of the scale ranged from 0·75 to 1·10, of Factor 2 from 0·87 to 1·08 and of Factor 3 from 0·74 to 1·34.

**Table 3:** The fit statistics of the C-PFS-T according to confirmatory factor analysis^(23,24)^

Fit indices	Cut-off criteria	C-PFS-T results
*χ*^2^/df	3–5	3·816
AGFI	≥0·90	0·931
RMR	≤0·05	0·063
RMSEA	0·06–0·08 or 1·00	0·082
GFI	0·85–1·00	0·852

AGFI, adjusted goodness-of-fit index; RMR, root mean square residual; RMSEA, root mean square error of approximation; GFI, goodness-of-fit index.

C-PFS-T: Turkish version of Children’s Power of Food Scale.

### Results of reliability analysis and test–retest analysis

According to Table 4, the Cronbach’s *α* internal consistency coefficient was 0·878 for the integrity of the C-PFS-T. The scale was reapplied to 18·6 % (*n* 50) of the sample number in order to reveal the scale’s invariance against time. All test–retest reliability coefficients were 0·784. Kappa# agreement coefficients and 95 % CI of *κ* agreement coefficients were given in Table 4, also. In this study food available, food tasted and total *κ* agreement coefficients were shown moderate; food present coefficients were shown a weak agreement.

**Table 4:** Reliability analysis and Cohen’s *κ* results concerning the subdimensions of the scale

C-PFS-T subdimensions	Item number	Cronbach’s *α* internal consistency coefficient	Test–retest reliability coefficients	Kappa# agreement coefficients	95 % CI of *κ* agreement coefficients
1. Food available	6	0·816	0·678	0·656	0·552, 0·721
2. Food present	4	0·709	0·413	0·572	0·459, 0·667
3. Food tasted	5	0·766	0·538	0·606	0·481, 0·719
Total	15	0·878	0·784	0·671	0·556, 0·739

C-PFS-T: Turkish version of Children’s Power of Food Scale.

# The coefficent of quadreting weighted *κ*.

The correlations of the items with the C-PFS-T total score ranged between 0·385 and 0·617, and Cronbach’s *α* coefficient if item deleted ranged between 0·866 and 0·877 (Table 5).

**Table 5:** Item-total scale correlation (*r*) and Cronbach’s *α* coefficient if item deleted

Subdimensions	Items	Item-total scale correlation (*r*)	Cronbach’s *α* coefficient if item deleted
Food available	1	0·556	0·869
	2	0·536	0·870
	5	0·560	0·869
	10	0·515	0·871
	11	0·573	0·868
	13	0·588	0·867
Food present	3	0·536	0·870
	4	0·385	0·877
	6	0·617	0·866
	7	0·463	0·873
Food tasted	8	0·601	0·867
	9	0·445	0·874
	12	0·593	0·867
	14	0·388	0·876
	15	0·596	0·867

C-PFS-T: Turkish version of Children’s Power of Food Scale.

In Table 6, C-PFS-T total score’s median values of normal weight group and obese group were 2·93 and 3·06, respectively.

**Table 6:** Total and subdimensions scores according to BMI groups (*n* 268)

	Normal-weight group (*n* 113)					Obese group (*n* 155)				
	min	max	Q1	Q2	Q3	min	max	Q1	Q2	Q3
C-PFS-T total score	1·20	4·73	2·20	2·93	3·46	1·27	5·00	2·53	3·06	3·66
Subdimensions										
Food available	0·40	2·00	0·73	1·00	1·33	0·40	2·20	0·80	1·06	1·46
Food present	0·27	1·33	0·60	0·80	1·00	0·33	1·33	0·73	0·93	1·13
Food tasted	0·33	1·67	0·80	1·06	1·33	0·33	1·67	0·86	1·06	1·33

C-PFS-T: Turkish version of Children’s Power of Food Scale; min: minimum; max: maximum; Q1: lower quartile; Q2: median; Q3: upper quartile.

## Discussion

The PFS is a simple and useful instrument that has been translated into several languages for measuring adults’ appetitive motives that precede food intake and consumption^(6,11,12,25)^. In addition, there are a limited number of studies using the child version of the scale^(13,14)^. The present research was the first attempt to validate the Turkish version of the C-PFS and to test the C-PFS-T in Turkish children and adolescents. In light of the validity and reliability studies conducted in different cultures and groups, we think that these scales can measure appetitive motives, identify children with a tendency for obesity and provide intercultural comparisons. For this purpose, 268 children and adolescents aged 9–16 years answered the C-PFS-T. The Turkish form consisted of three subdimensions and the total variance ratio was 54·7 %. The Cronbach’s *α* internal consistency coefficient was 0·878. Moreover, the test–retest reliability appeared to be adequate. The statistical results obtained from this scale showed the acceptability and applicability of the C-PFS-T in terms of the *χ*^2^/df, AGFI, RMSEA and GFI fit indices.

The internal consistency of the C-PFS-T was evaluated by Cronbach’s *α* internal coefficients. This value should be as close to 1 as possible. In the literature, if this value is between 0·60 and 0·80, the scale is quite reliable, and if it is between 0·80 and 1·00, the scale is highly reliable^(26)^. In the present study, the Cronbach’s *α* internal consistency coefficient was 0·878, and the subdimensions coefficients were 0·816, 0·709 and 0·766 for the food available, food present and food tasted factors, respectively. Moreover, in the present study, the overall Cronbach’s *α* internal consistency and food available subdimension had a high internal consistency, while the other subdimensions were quite reliable. In a study on the C-PFS, Laurent (2015) included adolescents aged 10–14 and examined the psychometric properties of the scale. The subgroup internal consistency coefficients ranged between 0·61 and 0·89^(13)^. The Cronbach’s *α* values of the subdimensions cannot be discussed since they are not given separately in that article. In the development and validation study of the nine-item short form of the C-PFS, it was stated that the internal consistency of the scale was excellent (*α* = 0·94) and the Cronbach’s *α* internal consistency coefficients of the subdimensions were 0·86–0·90^(27)^. In the Japanese validation study of the PFS in young adults, Cronbach’s *α* was 0·87 and the coefficients of subdimensions were 0·79–0·66^(14)^.

In the present study, according to test–retest reliability, the C-PFS-T has overall good reliability (0·784), but the subdimensions in particular have moderate to poor reliability (0·413–0·678). Moreover, the food present subdimension coefficient is lower than the others, thus reducing the overall test–retest coefficient. This may have been due to the 2-week interval between test and retest reliability. According to Streiner and Norman (2008), the appropriate time interval depends on the construct to be measured and the target population; however, approximately 2 weeks is often considered generally appropriate^(21)^. In addition in the original study of C-PFS^(13)^, procedures were repeated 16 (*v*. 14) days following the initial administration. In the same study, Laurent found that similar or lower test–retest reliability coefficients (overall 0·55; subdimensions 0·28–0·68) of the C-PFS compared to our study. In Laurent’s study, the food present subdimension had the lowest test–retest reliability coefficient also. This may have been due to the socio-economic status and multiethnicity of the participants^(13)^. In another study, in which the procedures were repeated after 3 weeks, the scale stability over time was excellent (overall 0·76)^(14)^.

DeVellis recommended that the structure should be determined by explanatory factor analysis with CFA^(19)^. According to *χ*^2^/df, the scale had a perfect fit. In addition, the AGFI was 0·931, and this indicated that the model was a good fit. The RMSEA value was 0·082, indicating adequate fit. The GFI value was 0·852, which showed a good fit. The CFA results were consistent with the criteria specified in the literature^(23,24)^. The statistical results obtained from this scale showed the acceptability and applicability of the C-PFS-T. In Laurent’s study, which determined the reliability and validity of the C-PFS for use in school age children, CFA was not carried out, so the results could not be compared. In a study that used the PFS for adolescents, the three-factor structure model fit statistics (RMSEA = 0·033, CFI = 0·985) indicated acceptable fit. In another study, a validation study of the C-PFS for use in Japanese children, the fit statistics for the model were as follows: *χ*^2^/df = 2·27, GFI = 0·87, AGFI = 0·82 > 0·80, RMSEA = 0·09 < 1·00, AIC = 263·6 and CFI = 0·86)^(14,28)^.

In the present study, C-PFS-T total score’s median values of normal weight group and obese group were 2·93 and 3·06, respectively. There was a weak, positive and significant correlation between BMI and C-PFS-T total score (*r* = 0·158, *P* = 0·010), food available score (*r* = 0·189, *P* = 0·002) and food present score (*r* = 0·166, *P* = 0·006) (data not shown). Similarly, in Hayzaran and Akçil Ok’s study (2020) conducted with students, there was a positive and statistically significant correlation between BMI and PFS scale score and food available score^(25)^. In contrast, in another study, a negative correlation was found between C-PFS short-form score and BMI^(27)^. Likewise, in the study by Mason *et al.* (2019), there was a negative correlation between adolescents’ initial C-PFS scores and their BMI a year later^(29)^. In the study by Mitchell *et al.*, no significant correlation was found between the C-PFS score of adolescents and pre-adolescents and their BMI^(30)^. These differences may have been due to individual differences and hedonic hunger only affecting BMI in the presence of impulsivity or some other psychological stimuli. Thus, clinical studies will be needed to determine whether hedonic hunger could change with time and affect the future BMI.

### Limitations

Despite its many strengths, there are some limitations of the present study. Concurrent validity analysis could not be conducted since there is no other parallel scale measuring hedonic hunger in children and adolescents. A cut-off point could not be determined to identify children with and without risk of hedonic hunger. Conclusions concerning a cause-and-effect relationship cannot be drawn due to the cross-sectional nature of the data in terms of BMI and C-PFS-T total score.

## Conclusions

Desire to consume palatable foods to experience pleasure beyond homoeostatic hunger is known as hedonic hunger and it is a phenomenon of interest in paediatric populations. Because of the rapid rise in childhood obesity, a tool that measures individual differences in appetite-related thoughts, feelings and motivations in an obesogenic environment can be useful for identification of groups susceptible to the development of obesity. A scale validated for Turkish children is needed in order for healthcare providers to establish a common assessment method. This study represents a step towards filling this gap in the paediatric assessment literature.

Finally, it was concluded that the Turkish version of the C-PFS, which provides an assessment of the hedonic hunger status of children and adolescents with fifteen items and three subdimensions, has sufficient reliability and validity to be applied to these subjects.

## Supplementary material

For supplementary material accompanying this paper visit http://dx.doi.org/10.1017/S1368980021003773. click here to view supplementary material
